# Successful Elimination of Gallbladder Ascariasis by Conservative Therapy, Followed by Cholecystectomy due to Developing Cholecystitis

**DOI:** 10.1155/2018/5831257

**Published:** 2018-12-10

**Authors:** Ahmad Alhamid, Ziad Aljarad, Ahmad Ghazal, Ahmad Mouakeh, Ahmad Sankari Tarabishi, Majed Joudeh, Mohammad Mohammad, Aos Alhamid, Jawhar Aljarad, Maen Mousa

**Affiliations:** ^1^Medical Student, Faculty of Medicine, University of Aleppo, Aleppo, Syria; ^2^Gastroenterologist in the Department of Internal Medicine, Faculty of Medicine, Aleppo University Hospital, University of Aleppo, Aleppo, Syria; ^3^Surgery Department, Faculty of Medicine, Aleppo University Hospital, University of Aleppo, Aleppo, Syria; ^4^Radiologist in the Department of Radiology, Faculty of Medicine, Aleppo University Hospital, University of Aleppo, Aleppo, Syria; ^5^Medical Student, Faculty of Medicine, Syrian Private University, Daraa, Syria; ^6^Gastroenterology Department, Faculty of Medicine, Aleppo University Hospital, University of Aleppo, Aleppo, Syria

## Abstract

**Background:**

* Ascaris lumbricoides* is the most common parasitic infection in human. The worm is usually located in the small intestine, but may invade into the pancreatic or biliary tree, but rarely into gallbladder because of the anatomic features of the cystic duct.

**Case Presentation:**

We report a case of gallbladder ascariasis that was diagnosed incidentally in a 70-year-old man, with negative ova and parasite test and no eosinophilia. We also compared echography and computerizied tomograph as diagnostic tools for gallbladder ascariasis. The patient was managed conservatively, but he underwent cholecyctectomy later because of developing cholecystitis.

**Conclusion:**

Depending on this case, we suggest cholecyctectomy as an initial management of gallbladder ascariasis.

## 1. Background

*Ascaris lumbricoides*, or roundworm, is considered as the most common parasitic infection worldwide [1]. The worm commonly lives in the jejunum and midsection ileum. The majority of intestinal infections are asymptomatic [2]. Invasion of the biliary tree by* Ascaris* is prevalent in endemic areas, where ascariasis is equal to gallstones as a cause of biliary diseases. GB ascariasis may cause cholecystitis, cholangitis, biliary colic, severe pancreatitis, and hepatic abscess [3]. However, gallbladder ascariasis (GB ascariasis) is quite rare because the cystic duct is narrow and tortuous, accounting for 2.1% of hepatobiliary ascariasis [4]. Ways of diagnosis include ultrasonography, magnetic resonance imaging (MRI), and endoscopic retrograde cholangiopancreatography (ERCP) [5]. GB ascariasis can be managed endoscopically or surgically [3], but it is rare to find reports of successful elimination of the worm by conservative therapy. In this article, we report a case of GB ascariasis that was discovered incidentally in a 70-year-old man. It was not accompanied with eosinophilia; no ova and parasites were detected in the feces. The worm was eliminated by conservative therapy, but the patient later underwent cholecystectomy because of developing cholecyctitis.

## 2. Case Presentation

A 70-year-old man presented to Aleppo University Hospital in order to have a routine check-up for benign prostatic hyperplasia (BPH) that was diagnosed when he was 31 years old.

**Figure 1:**
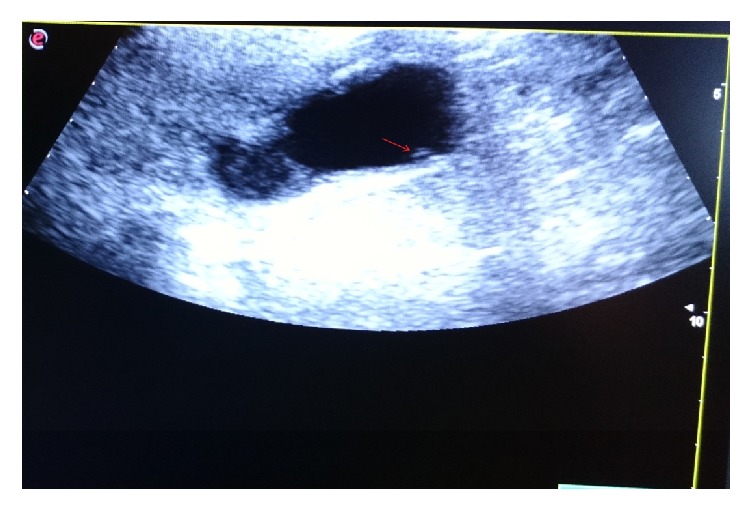
The* Ascaris lumbricoides* appearing as an echogenic, tubular-shaped, and coiled structure, with anechoic central line (arrow), located in the gallbladder.

Through investigations, we performed an echography of the abdomen and detected a tubular-shaped, moving, echogenic structure with anechoic central line located in the gallbladder; the thickness of gallbladder wall was 4 mm. We did not detect any calculi (Figure 1). These findings suggest gallbladder ascariasis.

As a history, he had controlled hypertension, a repaired hiatal hernia, gastroesophageal reflux disease (GERD), hemorrhoids, and BPH. However, his general condition was good.

He was asymptomatic, but mentioned that two weeks ago he had experienced nausea, vomiting, hyperthermia, chills, and abdominal pain which started in the right hypochondrial region, radiated to the umbilical region, and lasted for 5 days. The patient did not report jaundice or a change in bowel habit. He dealt with these symptoms himself and took over-the-counter ciprofloxacin, metronidazole, augmentine, and paracetamol.

**Figure 2:**
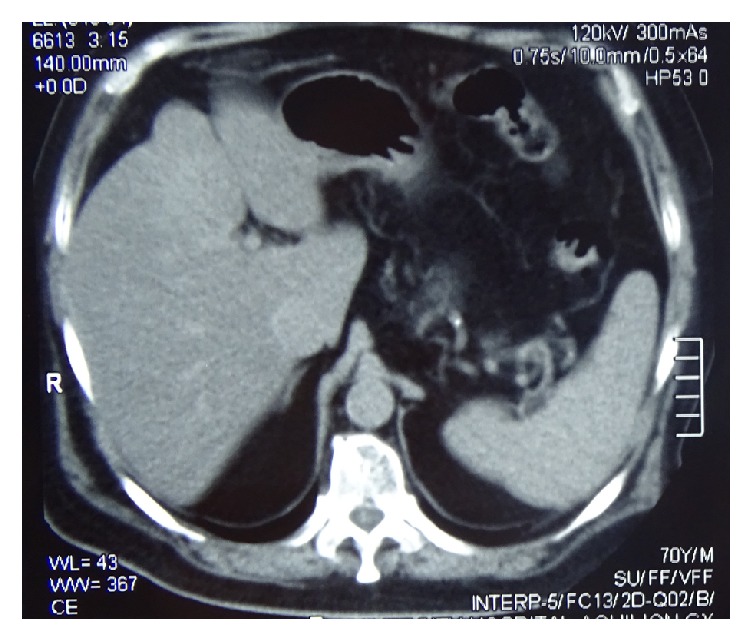
Multi-slice computerized tomography showing no worm even though the ultrasonography showed it.

Multi-slice computerized tomography (MSCT) have not shown any worm or calculi (Figure 2).

His laboratory findings were all normal including aspartate aminotransferase (AST), alanine aminotransferase (ALT), gamma glutamyl transferase (*γ*GT), bilirubin, C-reactive protein (CRP), erythrocyte sedimentation rate (ESR), amylase, and complete blood count (CBC). No eosinophilia existed. Ova and parasite (O&P) test was negative.

As the patient is an elderly and hypertensive and was so afraid from operation, conservative treatment was applied with albendazole 400 mg (single dose) and wide spectrum antibiotics, with observation on echography.

After one week, ecography revealed that the worm is still moving and so still alive; therefore, the patient was given a second dose of albendazole.

**Figure 3:**
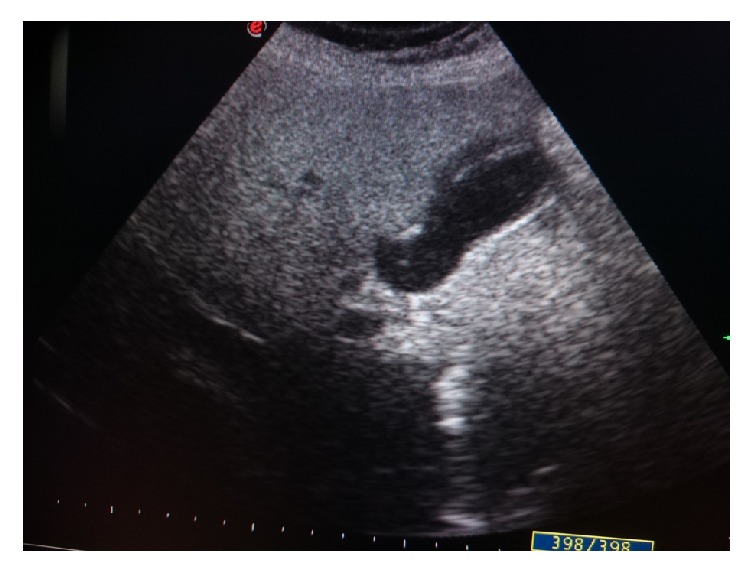
The echogenic debris in the gallbladder.

After 2 weeks, the worm has not appeared on echography, but we noticed an echogenic debris in the gallbladder (Figure 3).

So, the patient was advised to operate a cholecystectomy to avoid obstruction of bile or pancreatic ducts by the worm's debris, but he disapproved.

The patient returned back to hospital after 10 days; we checked his gallbladder with echography again. It was clear and we did not notice any debris, which means aspiration of the remains of the worm.

After 10 days, he presented to hospital complaining of nausea, vomiting, fever, jaundice, and right hypochondrial pain.

On echography, the common bile duct was not distended and no obstructing structure was noticed.

Laboratory tests revealed leuckocytosis (13800/mm^3^), with a left shift, as the neutrophils count was 12200/mm^3^. Eosinophils count was normal. We also found significant increase in CRP (20mg/l), mild elevation in ALT (115 U/L) and AST (90 U/L), and bilirubin was 4mg/dl.

A diagnosis of acute cholecystitis was made, and we perfomed cholecyctectomy for the patient.

There were many adhesions around the gallbladder.

Pathology report confirmed acute cholecystitis. We detected no macroscopic or microscopic remains of the worm in the gallbladder. Under microscope, we noticed a marked number of eosinophils in the bile of the gallbladder.

After three months, our patient is in a good health and has no complications.

## 3. Discussion and Conclusion

The roundworm* Ascaris lumbricoides* is the most common helminthic infection; it infects more than 1 billion people worldwide. Infections are mostly asymptomatic. The most common presentation is small bowel obstruction due to the mass of worms which obstructs the lumen of the intestine [6].

The most common settlement of the worm is in the jejunum middle of small intestine [2].

Due to the narrow and tortuous structure of the biliary tract, it is rare for the helminth to invade into the gallbladder, constituting 2.1% of hepatobiliary ascariasis [4].

Infection of the intestine is generally asymptomatic. However, important complications may happen, such as ascending cholangitis, acute acalculous cholecystitis, obstructive jaundice, pancreatitis, liver abscesses, and septicemia, with the settlement of the parasite in the biliary tract ascending upwards from the intestines [3].

The clinical features of gallbladder ascariasis are not pathognomonic. They usually include fever, jaundice, abdominal pain, vomiting, hepatomegaly, and right upper quadrant tenderness [7]. Our patient complained of nausea, vomiting, fever, chills, and right hypochndrial pain two weeks before he came to his routine check-up for benign prostatic hyperplasia.

Biliary ascariasis does not have any characteristic laboratory or clinical features, so radiology plays a critical role in the diagnosis of a parasite in the biliary tree. Computed tomography (CT) and magnetic resonance imaging (MRI) are used in the diagnosis of hepatobiliary ascariasis. Endoscopic retrograde cholangiopancreatography (ERCP) is a diagnostic and therapeutic tool. However, ultrasonography (US) is still the method that is first used and most preferred, in the follow-up as well, due to its ease of applicability and the fact that it is inexpensive and noninvasive. In addition, US enables active movements of the worm to be displayed, which helps to identify whether it is alive, an important advantage over CT and MRI [5].

On ultrasound, the worms present in the gallbladder and common bile duct as a linear echogenic image with anechoic line in the middle that represents the alimentary tract of the worm. No acoustic shadow appears [8].

The findings of erratic, nondirectional, zigzag movements are characteristic of a live worm [9].

Ultrasonography showed a linear echogenic image without acoustic shadow which is characteristic of worm. Thickness of the gallbladder wall was 4 mm (Figure 1).

Multi-slice computerized tomography (MSCT) did not show the worm (Figure 2).

Eosinophilia and positive stool examination for ova and parasite (O&P) may help in diagnosis.

In our case, the patient's eosinophils count was normal and O&P test was negative.

As suggested in the available literature, initial therapy for gallbladder ascariasis should involve conservative treatment, unless an associated disease is present or a complication arises, because some patients can be treated conservatively [10] (Topal) (Gonen). However, most patients need cholecystectomy later, due to the failure of the conservative therapy [10]. Following the suggestions of the previous studies, and regarding the age and comorbidities of our patient, we started with conservative treatment by administration of albendazole 400 mg (single dose) orally with wide spectrum antibiotics. After one week of follow-up with ultrasonography, the worm was still alive, so a second dose of albendazole was administrated. After two weeks, the worm did not appear on echography, but an echogenic debris was noticed in the gallbladder.

The need for multidrug antiparasitic treatment in our case, or the poor response of* Ascaris* to medical treatment in most cases, is because less than 1% of the antiparasitic drugs are excreted in bile [10].

Indications for cholecystectomy in gallbladder ascariasis include failure of a spontaneous clearance of worms after conservative treatment, a dead worm inside the gallbladder, and worm associated with calculi [10].

So the patient was advised to conduct a cholecystectomy but he disapproved. But the echography revealed the aspiration of this debris ten days later.

After another ten days, he presented to hospital complaining of nausea, vomiting, fever, jaundice, and right hypochondrial pain. On echography, common bile duct diameter was normal, and no obstruction was detected. A diagnosis of acute cholecystitis was made, and emergency cholecystectomy was performed and there were many adhesions. Pathology report revealed acute cholecystitis with no macroscopic or microscopic worm remains in bile. There was marked number of eosinophils in the bile of the gallbladder under microscope.

Now, after 3 months of follow-up, the patient is in a good health and there are no complications.

As a conclusion, gallbladder ascariasis should be kept in minds of physicians and radiologists while assessing patients with cholecystitis, especially in epidemic areas. Gallbladder ascariasis can be discovered incidentally. Negative O&P test and the absence of eosinophilia do not exclude the diagnosis of gallbladder ascariasis. Under microscope, the bile of the gallbladder may contain large amount of eosinophils. Echography is better than CT in the detection of gallbladder ascariasis, as echography in our case showed the worm while the CT did not. In addition, echography is of low cost and shows the movements of the worm. Studies suggest starting treatment with conservative therapy. However, depending on our case, we suggest cholecystectomy as an initial therapy, because the success of conservative therapy did not protect the patient from developing cholecystitis later. Also, conservative therapy with albendazole led to the sequestration of the worm, which presents a high risk of developing ascending cholangitis. In addition, conservative therapy often fails, and most patients will need cholecystectomy later. We suggest that the upcoming published papers about gallbladder ascariasis should follow up the patients for enough time to study the incidence of cholecystitis among patients that were treated without cholecystectomy.

## Data Availability

The datasets used and/or analyzed during the current study are available from the corresponding author on reasonable request.

## Abbreviation

GB: Gallbladder aascariasis
MRI: Magnetic resonance imaging
ERCP: Endoscopic retrograde cholangiopancreatography
BPH: Benign prostatic hyperplasia
GERD: Gastroesophageal reflux disease
MSCT: Multi-slice computerized tomography
AST: Aspartate aminotransferase
ALT: Alanine aminotransferase
*γ*GT: Gamma glutamyl transferase
CRP: C-reactive protein
ESR: Erythrocyte sedimentation rate
CBC: Complete blood count
O&P: Ova and parasite
CT: Computed tomography
US: Ultrasonography.
